# Environmentally Driven Migration in a Social Network Game

**DOI:** 10.1038/srep12481

**Published:** 2015-07-28

**Authors:** Masanori Takano, Kazuya Wada, Ichiro Fukuda

**Affiliations:** 1 CyberAgent, Inc., Chiyoda-ku, Tokyo, Japan

## Abstract

Cooperative behaviors are common in humans, and they are the fundamental basis of our society. Theoretical and experimental studies have modeled environments where the behaviors of humans, or agents, have been restricted to analyze their social behavior. However, it is important that such studies can be generalized to a less restrictive environment in order to understand human society. Social network games (SNGs) provide a powerful tool for the quantitative study of human behavior using a less restrictive environment than in previous studies. We focused on multilevel selection, one of the theoretical frameworks used to study the evolution of cooperation. The evolution of cooperation by multilevel selection requires that the continual assortment between cooperators and noncooperators is generated; thus, humans may have acquired mechanisms that ensure assortment (e.g., migration between groups). This study aims to investigate this mechanism in a less restrictive environment. For this purpose, we researched the effect of migration based on data analysis in an SNG where the players could behave more freely than was possible in the environments used in the previous studies. We showed that players maintained assortment between cooperators and defectors in this SNG, where it appears that environmentally driven migration generated the assortment.

Cooperative behaviors are common in humans, and they are fundamental basis of our society^1^,^2^. However, noncooperators obtain more advantages than cooperators during interactions, because cooperators would be exploited by noncooperators^3^; thus, natural selection should favor noncooperators. Nevertheless, humans cooperate with each other; therefore, they must have acquired mechanisms that ensure cooperation during the evolutionary process^4^.

These evolutionary dynamics require a structured interaction where cooperators interact more frequently with cooperators and noncooperators interact more frequently with defectors, because cooperators would be exploited by noncooperators. Thus, humans must have acquired mechanisms for assortment between cooperators and noncooperators during the evolutionary process. Five theoretical mechanisms have been proposed^5^: kin selection, direct reciprocity, indirect reciprocity, spatial selection, and multilevel selection. Theoretical and experimental studies have presented evidence of these mechanisms^6^. Evidence has been acquired using modeled environments with constrained behaviors of humans, or agents, to analyze their social behavior explicitly. However, it is important that this evidence be generalized to a less restrictive environment to understand human society^6^, because humans act motivated by various reasons including non-rational thinking^7^,^8^. These empirical findings (e.g.,^7^,^8^) would lead to new theoretical studies (e.g.,^9^).

Interactive online games are particularly powerful tools for the quantitative study of human society^10^,^11^,^12^,^13^,^14^. In online games, numerous players can behave more freely than is possible in the environments used in the theoretical and experimental studies^5^,^6^, i.e., they do not need to select from a sequence of several alternatives, because they always have multiple alternatives. In addition, the actions of all players can be recorded. In the present study, we analyzed a social network game (SNG), such as Rage of Bahamut (http://mobage.com/games/rage-of-bahamut) or Girl Friend BETA (http://vcard.ameba.jp) to understand cooperative behavior among humans, because the following features of SNGs provide easier analysis of cooperative behavior. SNGs allow real players to cooperate and compete with others in situations when the player’s benefit is represented by a quantitative value, such as a payoff in game theory.

The importance of co-evolution of individual strategy and population structure in the evolution of cooperation was shown by the theoretical studies^15^ which often used structured population models, e.g., subdivided groups, lattice networks, complex networks, and so on^16^,^17^,^18^. We focused on the theory of multilevel selection^19^,^20^ which is also one of the structured population models, where we considered a population that was subdivided into groups. An individual in a group that includes many cooperators obtains a higher payoff than one in a group that includes many noncooperators (between-group selection). At the same time, a noncooperator obtains a higher payoff than a cooperator within the group (within-group selection). Multilevel selection theory shows that cooperators evolve when the former type of selection predominates over the latter type. However, noncooperators always increase in their groups via within-group selection. Thus, the evolution of cooperation by multilevel selection requires that continual assortment between cooperators and noncooperators is generated; therefore, humans may have acquired mechanisms that facilitate assortment^21^.

If we consider migration between groups as the mechanism, unintelligent migration decreases the assortment, i.e., all individuals leave and enter the group with equal probability, and frequent random migration reduces the variation between groups^20^,^22^. On the other hand, intelligent migration generates assortment^23^,^24^,^25^. Ichinose and Arita^25^ estimated the effect of migration on the evolution of cooperation in an individual-based simulation. In their model, individuals migrated between groups in response to a poor environment. This behavior is referred to as environmentally driven migration. The model showed that cooperators often migrated from a group that included many noncooperators because their payoff was lower than the noncooperators’ payoff. The simulation demonstrated that environmentally driven migration can generate a biased distribution between groups. Additionally, there are other partner selection models, e.g., spatial moves^26^, dynamical social networks^27^,^28^, parasite migration from hosts in host-parasite relationships^29^, and so on. A time scale difference between individual strategy and population structure, which was a control parameter, was critical for emergence of cooperative behavior in these studies^25^,^26^,^27^,^28^,^29^.

The behavior when selecting partners, which is similar to environmentally driven migration, was confirmed in humans by the experimental studies^30^,^31^,^32^. Wang *et al.*^32^ showed that the selection of partners promotes cooperation in the prisoner’s dilemma game using a social network. Players maintained the link with their partner when they were satisfied (i.e., their payoff was higher than that of their game partner). However, the players often broke the link with their partner if they were not satisfied (i.e., their payoff was smaller than that of their game partner). Thus, a cooperator broke their links with noncooperators and made links with others in the same manner as environmentally driven migration. As a result, cooperators interacted more frequently with cooperators and noncooperators interacted more frequently with noncooperators. A time scale difference between individual strategy and partner selection was also discussed in these experimental studies. In the studies^31^,^32^, the maintenance of cooperation required that the time scale of partner selection was short. On the other hand, the maintenance of cooperation was possible even if individuals did not select partners (i.e., the time scale of partner selection was very long)^33^. The previous theoretical^25^,^26^,^27^,^28^,^29^ and experimental^31^,^32^,^33^ studies discussed a time scale difference between individual strategy and partner selection was critical for the emergence of cooperative behavior.

This study aims to investigate this mechanism that generates the assortment between cooperators and noncooperators in a less restrictive environment. For this purpose, we research the effect of migration on the assortment based on data analysis in an SNG where the players can behave more freely than is possible in the environments used in the previous studies, e.g., they can update their partners anytime. We focus on a game scenario where the cooperative behavior is defined by constructing a payoff matrix in the situation. Based on this definition, we analyze players’ cooperative behavior and their migration behavior.

## Materials and Methods

In this section, we provide the minimal SNG information and the definition of cooperative behavior in an SNG (see Appendix section A, B, and C for game information, rules, and definition, respectively).

We analyzed cooperative behavior in the SNG, “Girl Friend BETA,” in which players acquired “event points” and competed in the rankings based on those points, because the players received better awards as their rankings increased. This SNG released at 29/10/2012. The player’s ranking order was determined by the sum of event points obtained in the period from 3/25/2013 to 4/8/2013. It was impossible to analyze societal dynamics in this SNG, because the rules changed frequently. The situation in the SNG was also unstable in the early stage of this period; therefore, for simplification, we used only the data from the final three days.

The event points for players’ actions correlate significantly with their levels, one of a player’s attributes (accurately, the event points per player’s action depend primarily on players’ attack power, which strongly correlates with their levels. The game did not store to a log file, hence we used players’ levels as alternatives). Players must spend their energy to obtain event points; therefore, the number of players’ actions is finite. There are two methods for replenishing these points, waiting for the points to replenish over time and using a paid item. Let “payment amount” be the sum of money spent by each player during the analysis period. Players must use their resources (items and time) effectively to progress to a higher ranking, because any player’s time and money are finite.

Players belong to groups in which they cooperate with each other to play the game efficiently; the groups were limited to 1–50 players. The SNG is designed to ensure that cooperation with group members results in an effective game play. We filtered out players who do not belong to groups, because almost all active players belonged to groups to play effectively. Active players can create groups on their own. Others can apply to join groups at any time and then join a group after acceptance of the application by an administrator, who is typically a group founder. Players can leave a group at any time and apply to join a different group. We regarded this behavior as migration. Players observe their group members’ behavior (e.g., attack on common enemies (see details later)), because the game system shows their behavior on the game screen. We targeted groups of five or more active players who logged in at least one or more times to analyze social interactions.

Players can communicate at any time through simple text messaging. This does not negatively affect either senders or receivers; nevertheless, its positive effects are also few. Players acquire a few points for a lottery that provides a card, when the players send messages to other at the beginning of each day. However, players must pay 200 points for the lottery, and the effect of the card is small, i.e., the points do not increase players’ abilities.

We analyzed cooperative behavior based on migration in the above environment. Table 1 shows its basic numerical information. The proportion of migrators was 0.224. It was difficult to track all cooperative behavior, because players can exhibit various behaviors in the SNG. Hence, we selected a specific cooperative behavior among various cooperative behaviors and regarded the frequency of that behavior as a measure of a player’s cooperativeness.

We focused on a game scenario in which the relationship between players was similar to that in the Leader game (Table 2), but it was not possible for both players to cooperate at the same time in this scenario (see appendix C). In the Leader game, Pareto efficiency is achieved, when one player cooperates and the other does not. Then the cooperator receives *S*, and the noncooperator *T*. However, both try to avoid the worst situation (i.e., they get *P*), but they also do not want to pay the cost to avoid the worst situation (i.e., they do not want to get *S*). That is, players receive a high payoff by sharing *S* and *T* on repeated plays of the game, a process known as *ST* reciprocity^34^. We recognized this cooperative behavior, which provides the payoff *T* from one to the other, as a cooperative behavior in this scenario.

Here, we consider the payoffs for cooperators and noncooperators in a population, which are terms used in the analysis. When the proportion of cooperators was *p* ∈ [0,1], the expected value of a cooperator’s payoff was as follows:


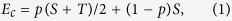


where the first term is the expected value when a cooperator interacts with other cooperators because either of the cooperators can attack an enemy. The second term is the expected value when a cooperator interacts with noncooperators because the cooperator always attacks an enemy. The expected value for a noncooperator’s payoff is as follows:


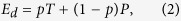


where the first term is the expected value when a noncooperator interacts with cooperators because the cooperators always attack an enemy. The second term is the expected value when a noncooperator interacts with noncooperators because the noncooperators do not attack an enemy.

## Results

**Figure 1:**
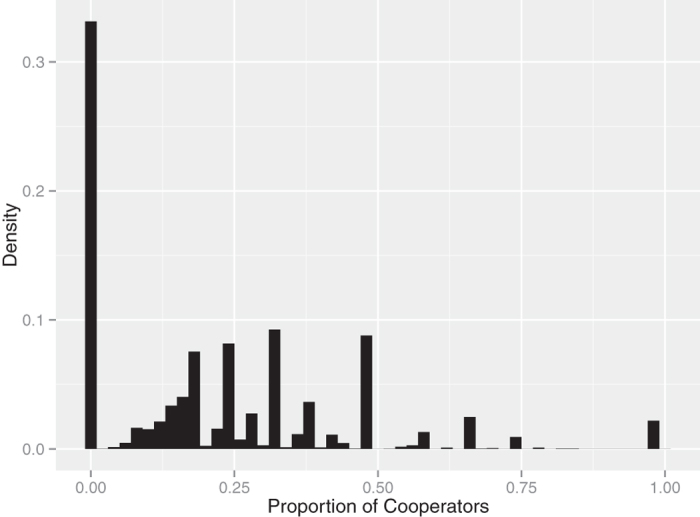
Density distribution of the proportion of cooperators in each group. There were two major classes of groups: one comprised only noncooperators and another comprised of both cooperators and noncooperators. Thus, assortment was generated between cooperators and noncooperators.

Figure 1 shows the density distribution for the proportion of cooperators in each group. There were two major classes of groups: one comprised only noncooperators (noncooperative group, group D) whereas cooperators coexisted with noncooperators in the other (cooperative group, group C). The coexistence of cooperators and noncooperators is trivial, because we defined cooperators based on the Leader game. On the other hand, it is interesting that assortment occurred between cooperators and noncooperators, because if players did not form group structures then the proportion of cooperators in each group should have been equivalent. We compared the two classes to analyze the cooperative behavior.

Why was this assortment generated? The expected value when a cooperator interacts with another cooperator is (*S* + *T*)/2. The expected value when a cooperator interacted with a noncooperator is *S* (and a noncooperator acquires *T*). Thus, the cooperators had to interact with other cooperators to acquire a high payoff since (*S* + *T*)/2 > S. Therefore, the cooperators probably formed groups with other cooperators to avoid noncooperators.

**Figure 2:**
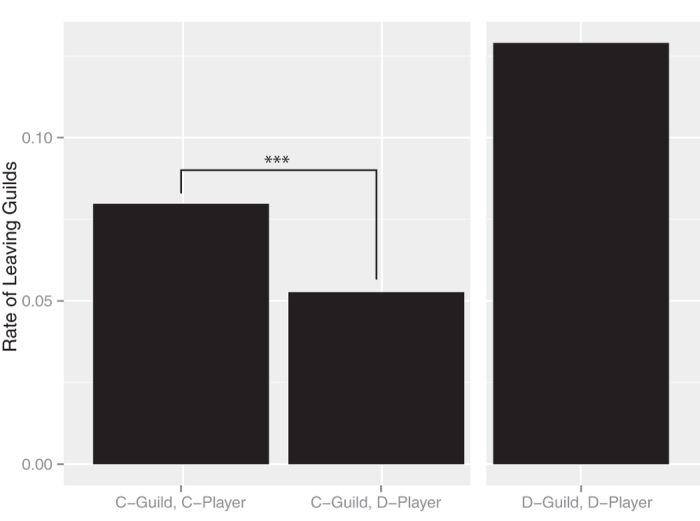
The rate of leaving groups. “C-group, C-Player” represents cooperators who belonged to cooperative groups. “C-group, D-Player” represents noncooperators who belonged to cooperative groups. “D-group, D-Player” represents noncooperators who belonged to noncooperative groups. ***indicates a significant difference at *P* = 8.5 × 10^−5^, chi-squared test. The number of “C-group, C-Players” was 1131 and the number of “C-group, D-Players” was 3869. We obtained a random sample of 5000 from all of the cooperative groups. The cooperators left the groups more frequently than the noncooperators.

How did the cooperators maintain their group structures? Figure 2 shows the rate at which members left groups, where the cooperators left the groups more frequently than the noncooperators. Thus, it appears that the cooperators searched for more cooperative groups, i.e., environmentally driven migration. As a result, the assortment between cooperators and noncooperators was generated.

**Figure 3:**
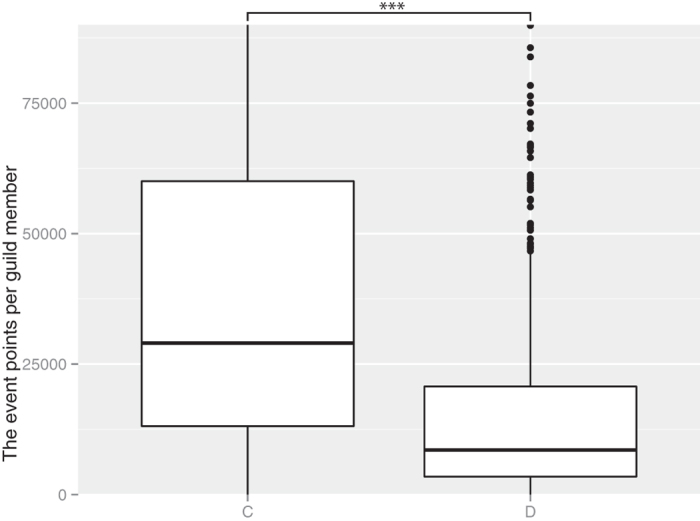
The event points per group member of cooperative groups (C) and noncooperative groups (D). The values shown were multiplied by a constant value to hide secret information and the same applies to the values in Figs 4, 5, 6. ***indicates a significant difference at *P* < 2.2 × 10^−16^, Wilcoxon’s rank-sum test. The number of C was 1683 and the number of D was 817. We obtained a random sample of 2500 from all of the groups. The members of the cooperative groups had more event points per member than did the noncooperative group members.

**Figure 4:**
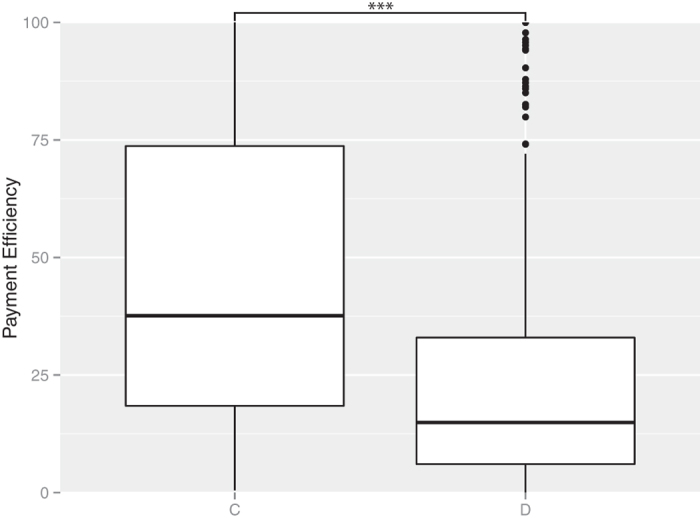
Payment efficiency for the cooperative groups and noncooperative groups. ***indicates a significant difference at *P* < 2.2 × 10^−16^, Wilcoxon’s rank-sum test. The number of C was 1683 and the number of D was 817. We obtained a random sample of 2500 from all of the groups. The payment efficiencies were higher for the cooperative groups than the noncooperative groups.

Here, we consider the payoff (payment efficiency) at the group level and the individual (player) level to estimate the effect of cooperative behavior based on evolutionary game theory. First, we consider the payment efficiency at the group level. Cooperative group members should obtain better benefits than noncooperative group members based on multilevel selection theory. Figures 3,4 show the mean of event points in each group and the mean of payment efficiencies in each group. In both cases, the cooperative groups had higher values than the noncooperative groups.

How did the cooperative groups obtain their advantages? The expected value of the payoff for a cooperative group (i.e., *p* had a maximum value of 1) was (*S* + *T*)/2. By contrast, the expected value of the payoff for a noncooperative group (i.e., *p* had a minimum value of 0) was *P*. Therefore the former situation was more advantageous than the later since (*S* + *T*)/2 > *P*. Thus, the players who belonged to cooperative groups increased their payoff via cooperation.

**Figure 5:**
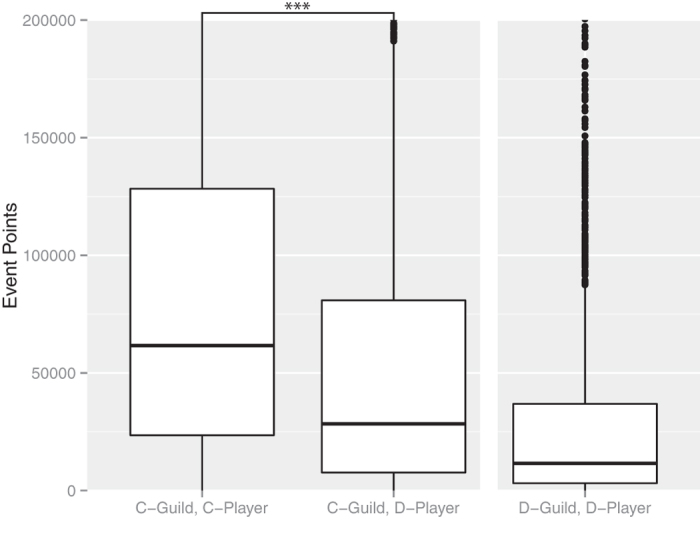
Event points of cooperators and noncooperators. ***indicates a significant difference at *P* < 2.2 × 10^−16^, Wilcoxon’s rank-sum test. The number of “C-group, C-Players” was 2134, the number of “C-group, D-Players” was 5362, and the number of “D-group, D-Players” was 2504. We obtained a random sample of 10000 from all of the players. The cooperators had more event points than the noncooperators.

**Figure 6:**
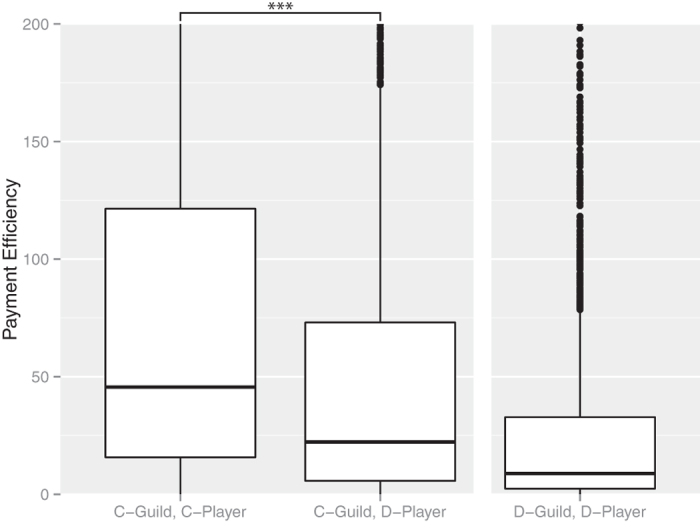
Payment efficiency for cooperators and noncooperators. ***indicates a significant difference at *P* < 2.2 × 10^−16^, Wilcoxon’s rank-sum test. The number of “C-group, C-Players” was 2289, the number of “C-group, D-Players” was 5276, and the number of “D-group, D-Players” was 2435. We obtained a random sample of 10000 from all players with payments. The payment efficiency of the cooperators was higher than that of the noncooperators.

Next, we consider the payment efficiency at the individual level in cooperative groups. In cooperative groups, noncooperators should obtain better benefits than cooperators based on multilevel selection theory. Figures 5 and 6 show players’ event points of players and the payment efficiencies of players. In both cases, the cooperative groups had higher values than the noncooperative groups.

How did the cooperators obtain their advantages? We consider the individual level in cooperative groups (i.e., *p* had a maximum value of 1). The expected value for a cooperator’s payoff was (*S* + *T*)/2. By contrast, the expected value for a noncooperator’s payoff was *T*. Therefore, the noncooperators should have obtained a higher payoff than cooperators. However the cooperators received a higher payoff than the noncooperators, as described above. It may have been due to that the equation (1) and (2) assume that intragroup interaction is homogeneous, because if the interaction was heterogeneous (i.e., players selected interaction partners in their groups) then cooperators may obtain better benefits than noncooperators.

**Figure 7:**
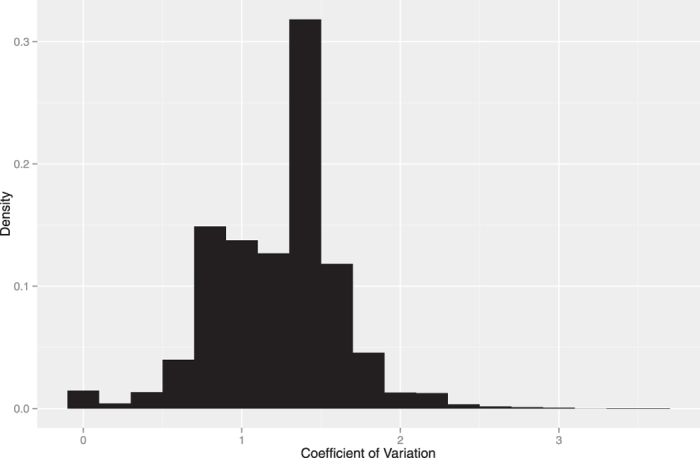
Density of coefficient of variation for cooperated frequency in each group.

Figure 7 shows the density histogram of the coefficient of variation for the number of cooperation from others to each player in each group. The most of values were far from 0, i.e., intragroup interactions were heterogeneous. This heterogeneity may suggest that other underlying mechanisms had effects (e.g., reciprocal altruism^35^,^36^).

## Summary

In the present study, we showed that players maintained assortment between cooperators and noncooperators in this SNG, where it appears that environmentally driven migration generated assortment. In addition, the cooperators played the game more efficiently.

The present study provides quantitative confirmation that the evidence described in the previous studies^25^,^27^,^28^,^31^,^32^ is applicable to an environment where players can behave with fewer restrictions than the previous modeling-based studies without controlling the frequency of partner selection which was discussed in the previous theoretical^25^,^26^,^27^,^28^,^29^ and experimental^31^,^32^,^33^ studies as a critical parameter for emergence of cooperative behavior. The frequency may have been in an intermediate range like the previous study^28^, because the proportion of migrators was reasonably far from 0 and 1, i.e., 0.224. In addition, our results suggest that the underlying cooperative behavior is based on other mechanisms (e.g., reciprocal altruism) because cooperators acquired more advantages than noncooperators in cooperative groups and their cooperative interactions were heterogeneous. The analysis of this phenomenon is a challenge for the future research.

In this study, we focused on migration behavior as a support mechanism for multilevel selection. However, in reality, humans use multiple mechanisms to cooperate with each other, e.g., migration, direct reciprocity, indirect reciprocity, and so on^5^,^6^, where time scale between strategy updating and partner selection based on these mechanisms seem to important^16^,^17^,^18^. Therefore, we would like to consider the interaction of these mechanisms and their time scale in our future work.

## Additional Information

**How to cite this article**: Takano, M. *et al.* Environmentally Driven Migration in a Social Network Game. *Sci. Rep.* **5**, 12481; doi: 10.1038/srep12481 (2015).

## Figures and Tables

**Table 1:** Basic numerical information of the analysis target.

Names	Value
The number of players	35,071
The number of groups	6,234
The number of migration players	7,863

**Table 2:** Payoff matrix of the leader game, where *S* + *T* > 2*R* and *T* > *S* > *R* > *P*. That is, Pareto efficiency is achieved, when one cooperates and the other does not cooperate.

	Cooperation	Noncooperation
Cooperation	*R*,*R*	*S*,*T*
Noncooperation	*T*,*S*	*P*,*P*

Then the cooperator obtains *S*, and the noncooperator *T*.

## References

[b1] Fehr E. & Fischbacher U. The Nature of Human Altruism. Nature 425, 785–791 (2003). 14574401 10.1038/nature02043

[b2] Smith J. M. & Szathmáry E. The Origins of Life: From the Birth of Life to the Origin of Language (Oxford University Press, 2000).

[b3] Axelrod R. The Evolution of Cooperation: Revised Edition (Basic Books, 2006).

[b4] Barkow J. The Adapted Mind: Evolutionary Psychology and the Generation of Culture (Oxford University Press, 1995).

[b5] Nowak M. A. Five Rules for the Evolution of Cooperation. Science 314, 1560–1563 (2006). 17158317 10.1126/science.1133755 PMC3279745

[b6] Rand D. G. & Nowak M. A. Human Cooperation. Trends in Cognitive Sciences 17, 413–425 (2013). 23856025 10.1016/j.tics.2013.06.003

[b7] Rand D. G. *et al.* Intuitive Cooperation and the Social Heuristics Hypothesis: Evidence from 15 Time Constraint Studies. SSRN Electronic Journal (2013).

[b8] Peysakhovich A. & Rand D. G. Habits of Virtue: Creating Norms of Cooperation and Defection in the Laboratory. SSRN Electronic Journal (2013).

[b9] Hoffman M., Yoeli E. & Nowak M. A. Cooperate without looking: Why we care what people think and not just what they do. Proceedings of the National Academy of Sciences of the United States of America 112, 1727–1732 (2015). 25624473 10.1073/pnas.1417904112 PMC4330730

[b10] Castronova E. On the Research Value of Large Games: Natural Experiments in Norrath and Camelot. Games and Culture 1, 163–186 (2006).

[b11] Bainbridge W. S. The Scientific Research Potential of Virtual Worlds. Science 317, 472–476 (2007). 17656715 10.1126/science.1146930

[b12] Szell M. & Thurner S. Measuring Social Dynamics in a Massive Multiplayer Online Game. Social Networks 32, 313–329 (2010).

[b13] Szell M., Sinatra R., Petri G., Thurner S. & Latora V. Understanding Mobility in a Social Petri Dish. Scientific Reports 2, 457 (2012). 22708055 10.1038/srep00457 PMC3375635

[b14] Szell M. & Thurner S. How Women Organize Social Networks Different from Men. Scientific Reports 3, 1214 (2013). 23393616 10.1038/srep01214 PMC3566601

[b15] Perc M. & Szolnoki A. Coevolutionary games-a mini review. Bio Systems 99, 109–25 (2010). 10.1016/j.biosystems.2009.10.003 19837129

[b16] Szabó G. & Fáth G. Evolutionary games on graphs. Physics Reports 446, 97–216 (2007).

[b17] Roca C. P., Cuesta J. A. & Sánchez A. Evolutionary game theory: Temporal and spatial effects beyond replicator dynamics. Physics of life reviews 6, 208–49 (2009). 20416850 10.1016/j.plrev.2009.08.001

[b18] Perc M., Gómez-Gardeñes J., Szolnoki A., Flora L. M. & Moreno Y. Evolutionary dynamics of group interactions on structured populations: a review. Journal of the Royal Society, Interface / the Royal Society 10, 20120997 (2013). 10.1098/rsif.2012.0997 PMC3565747 23303223

[b19] Wilson D. S. A theory of group selection. Proceedings of the National Academy of Sciences 72, 143–146 (1975). 10.1073/pnas.72.1.143 PMC432258 1054490

[b20] Traulsen A. & Nowak M. A. Evolution of Cooperation by Multilevel Selection. Proceedings of the National Academy of Sciences of the United States of America 103, 10952–10955 (2006). 16829575 10.1073/pnas.0602530103 PMC1544155

[b21] Fletcher J. A. & Zwick M. Strong altruism can evolve in randomly formed groups. Journal of theoretical biology 228, 303–13 (2004). 15135029 10.1016/j.jtbi.2004.01.004

[b22] Ono S., Misawa K. & Tsuji K. Effect of Group Selection on the Evolution of Altruistic Behavior. Journal of Theoretical Biology 220, 55–66 (2003). 12453450 10.1006/jtbi.2003.3144

[b23] Pepper J. W. & Smuts B. B. A mechanism for the evolution of altruism among nonkin: positive assortment through environmental feedback. The American naturalist 160, 205–13 (2002). 10.1086/341018 18707487

[b24] Pepper J. W. Simple models of assortment through environmental feedback. Artificial life 13, 1–9 (2007). 17204009 10.1162/artl.2007.13.1.1

[b25] Ichinose G. & Arita T. The Role of Migration and Founder Effect for the Evolution of Cooperation in a Multilevel Selection Context. Ecological Modelling 210, 221–230 (2008).

[b26] Chen X., Szolnoki A. & Perc M. Risk-driven migration and the collective-risk social dilemma. Physical Review E 86, 036101 (2012). 10.1103/PhysRevE.86.036101 23030974

[b27] Santos F., Pacheco J. & Lenaerts T. Cooperation prevails when individuals adjust their social ties. PLoS computational biology 2, e140 (2006). 17054392 10.1371/journal.pcbi.0020140 PMC1617133

[b28] Chen X., Fu F. & Wang L. Social tolerance allows cooperation to prevail in an adaptive environment. Physical Review E 80, 051104 (2009). 10.1103/PhysRevE.80.051104 20364944

[b29] Damore J. A. & Gore J. A slowly evolving host moves first in symbiotic interactions. Evolution; international journal of organic evolution 65, 2391–8 (2011). 10.1111/j.1558-5646.2011.01299.x PMC3145917 21790584

[b30] Fehl K., van der Post D. J. & Semmann D. Co-evolution of behaviour and social network structure promotes human cooperation. Ecology letters 14, 546–51 (2011). 21463459 10.1111/j.1461-0248.2011.01615.x

[b31] Rand D. G., Arbesman S. & Christakis N. Dynamic Social Networks Promote Cooperation in Experiments with Humans. Proceedings of the National Academy of Sciences 108, 19193–19198 (2011). 10.1073/pnas.1108243108 PMC3228461 22084103

[b32] Wang J., Suri S. & Watts D. J. Cooperation and assortativity with dynamic partner updating. Proceedings of the National Academy of Sciences of the United States of America 109, 14363–8 (2012). 22904193 10.1073/pnas.1120867109 PMC3437882

[b33] Rand D. G., Nowak M. A., Fowler J. H. & Christakis N. A. Static network structure can stabilize human cooperation. Proceedings of the National Academy of Sciences of the United States of America 111, 17093–17098 (2014). 25404308 10.1073/pnas.1400406111 PMC4260616

[b34] Tanimoto J. & Sagara H. Relationship between dilemma occurrence and the existence of a weakly dominant strategy in a two-player symmetric game. Biosystems 90, 105–114 (2007). 17188808 10.1016/j.biosystems.2006.07.005

[b35] Lindgren K. Evolutionary Phenomena in Simple Dynamics. In Artificial Life II 295–312 (1991).

[b36] Nowak M. A. & Sigmund K. A Strategy of Win-Stay, Lose-Shift That Outperforms Tit-for-Tat in the Prisoner’s Dilemma Game. Nature 364, 56–58 (1993). 8316296 10.1038/364056a0

